# Preventive Effects of Anthraquinones Isolated from an Endophytic Fungus, *Colletotrichum* sp. JS-0367 in Tumor Necrosis Factor-α-Stimulated Damage of Human Dermal Fibroblasts

**DOI:** 10.3390/antiox10020200

**Published:** 2021-01-30

**Authors:** Sullim Lee, Quynh Nhu Nguyen, Hung Manh Phung, Sang Hee Shim, Daeyoung Kim, Gwi Seo Hwang, Ki Sung Kang

**Affiliations:** 1 Department of Life Science, College of Bio-Nano Technology, Gachon University, Seongnam 13120, Korea; sullimlee@gachon.ac.kr (S.L.); davekim@gachon.ac.kr (D.K.); 2 College of Korean Medicine, Gachon University, Seongnam 13120, Korea; nnquynh173@gmail.com (Q.N.N.); 201940218@gc.gachon.ac.kr (H.M.P.); seoul@gachon.ac.kr (G.S.H.); 3 College of Pharmacy, Duksung Women’s University, Seoul 01369, Korea; sangheeshim@duksung.ac.kr

**Keywords:** skin aging, ROS, TNF-α, human dermal fibroblasts, anthraquinone

## Abstract

Reactive oxygen species (ROS) are a major causative factor of inflammatory responses and extracellular matrix degradation. ROS also cause skin aging and diverse cutaneous lesions. Therefore, antioxidants that inhibit the generation of ROS may be beneficial in the relief of skin aging and diseases. We investigated the anti-skin aging effect of anthraquinones from cultures of *Colletotrichum* sp., an endophytic fungus isolated from *Morus alba* L. using human dermal fibroblasts (HDFs). We preferentially evaluated the preventive effects of anti-oxidative anthraquinones (**1**, **4**) against the generation of ROS, nitric oxide (NO), and prostaglandins-E_2_ (PGE_2_). Among them, 1,3-dihydroxy-2,8-dimethoxy-6-methylanthraquinone (**1**) suppressed the generation of ROS, NO, and PGE_2_ in tumor necrosis factor-alpha (TNF-α)-stimulated HDFs. Compound **1** reversed the TNF-induced increase in matrix metalloproteinase (MMP)-1 and a decrease in procollagen I α1 (COLIA1). It also suppressed inducible NO synthase, cyclooxygenase-2, interleukin (IL)-1β, IL-6, and IL-8, which upregulate inflammatory reactions. Mechanistically, compound **1** suppressed nuclear factor-κB, activator protein 1, and mitogen-activated protein kinases in TNF-α-stimulated HDFs. These results suggest that compound **1** may be beneficial for improving skin aging and diverse cutaneous lesions.

## 1. Introduction

The skin is the primary protective organ of the human exodermal system and is in direct contact with potentially harmful factors. Because of its direct exposure to the external environment, the skin is the most prominent tissue affected by aging and damage [1,2]. Intrinsic aging involves damage that occurs with time due to decreased skin cell activity by reactive oxygen species (ROS) produced during skin cell metabolism [3]. Extrinsic aging is induced by exposure to external environmental hazards, such as pollution, chemicals, smoking, and ultraviolet (UV) radiation [4,5,6].

UV radiation is a key extrinsic stimulator. It mainly results in cumulative skin damage, referred to as photoaging [2]. UV radiation is classified based on wavelength into three categories UV-A, B, C. UVA and UVB comprise the entire UV spectrum on Earth. These wavelengths pass through the Earth’s atmospheric layers to the surface and can damage living things [7]. Exposure of the skin to UV radiation in everyday life produces a variety of physiological effects that include sunburn, photoaging, skin pigmentation, and production of ROS [8].

The production of ROS has been reported to cause oxidative damage to biological functions. The damage includes cell membrane destruction, DNA denaturation, and inflammatory response and immunodeficiency [9,10,11]. ROS are generated by oxidative phosphorylation in the mitochondria. Detrimental foreign material can induce the generation of ROS [12]. In skin, excess ROS forms wrinkles through chain crossing errors and cleavage of fibrous proteins including collagen and elastic fibers, which comprise the extracellular matrix (ECM) of the skin [4]. Collagenases like matrix metalloproteinase-1 (MMP-1) inhibit collagen synthesis [13]. The main cause of extrinsic aging is an ROS-mediated secondary reaction that occurs when UV radiation is absorbed by the skin [14]. Antioxidants that inhibit the generation of ROS and MMP-1 may potentially be beneficial in skin aging.

UV radiation causes an inflammatory response in the skin, leading to skin photoaging [15,16]. The light can directly or indirectly induce several proinflammatory mediators that include prostaglandin E_2_ (PGE_2_), cyclooxygenase-2 (COX-2), inducible nitric oxide synthase (iNOS), tumor necrosis factor-alpha (TNF-α), interleukin (IL)-1β, IL-6, and IL-8 [17,18,19]. In particular, these inflammatory responses are also associated with damage to skin fibroblasts, which accelerates the UV irradiation-induced photoaging process [17,20].

In addition, UV exposure of the skin can stimulate aging-promoting enzymes, and MMPs, such as collagenase (MMP-1) and elastase [14]. Elevated MMPs are responsible for the deterioration of the ECM components and other basement membrane constituents. Collagen, elastin, and ECM proteins form organized structures and serve as the primary constitutive framework that provides tensile strength to the skin [21,22]. Accordingly, the degradation of collagen and elastin can lead to premature and undesired appearance characteristics that include deep wrinkles, sagging, drooping, and atrophied skin [23]. Several studies on alleviating wrinkles in skin focused on the inhibitory activity of collagenase and elastase [24,25].

Root bark and leaves of *Morus alba* L. (mulberry) have been utilized in traditional oriental medicine and the sericulture industry [26,27,28]. Extracts of leaves and root bark reportedly display bioactivities that include hypoglycemic [29,30], antidiabetic [31], antiobesity [32], hepatoprotective [33] and anti-inflammatory effects.

Endophytic fungi and the host plant have a symbiotic relationship, producing endophytes [34]. Endophytes are considered potential drug candidates due to their diverse biological activities [35,36]. Extracts and secondary metabolites of *Colletotrichum* sp. with genetic diversity have been reported to have antimicrobial activity [37].

**Figure 1:**
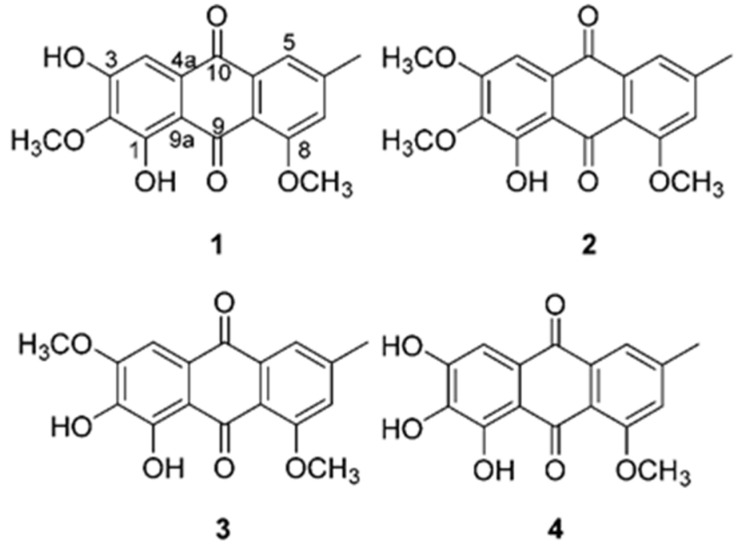
Structure of compounds **1**–**4**.

In a previous study, we isolated and structurally characterized four anthraquinones, including a novel anthraquinone, from cultures of *Colletotrichum* sp., an endophytic fungus isolated from *M. alba* L. (Figure 1). We also we evaluated their neuroprotective effects in glutamate-stimulated murine HT22 hippocampal neuronal cell line. Mechanistically, the most potent anthraquinone (evariquinone) prevented the generation of ROS, intracellular calcium ion levels, and phosphorylation of mitogen-activated protein kinases (MAPKs) in glutamate-mediated apoptosis [38]. Numerous studies have demonstrated that the antioxidant effect of anthraquinones may suppress oxidative stress within cells.

The foregoing indicates that anthraquinones should prevent oxidative stress related damage to the skin ECM. To explore this, we investigated the prevent effect against skin damages of anthraquinones obtained from *Colletotrichum* sp. using human dermal fibroblasts (HDFs) to identify potential candidates. Presently, we describe the prevent effect of skin damages of anti-oxidative anthraquinones and verify the mechanism of the active compound for TNF-α-stimulated HDFs.

## 2. Materials and Methods

### 2.1. Cell Culture and Treatment

HDFs purchased from PromoCell GmbH (Sickingenstr, Heidelberg, Germany) were maintained in Dulbecco’s modified Eagle’s medium (Corning, Manassas, VA, USA) supplemented with 10% heat-inactivated fetal bovine serum (Atlas, Fort Collins, CO, USA), 1% antibiotics (100 unit/mL of penicillin and 100 µg/mL of streptomycin; Gibco, Grand Island, NY, USA) at 37 °C in a humid atmosphere of 5% CO_2_ in an incubator.

The cells were seeded in each size well plate at a density of 3 × 10^4^ cells/cm^2^ and allowed to adhere. The media were subsequently changed with serum-free medium and incubation was continued until analysis. A stock solution (20 µg/mL) of TNF-α (PeproTech, Rocky Hill, NJ, USA) was prepared. The solution also contained 1% bovine serum albumin (Sigma-Aldrich, St. Louis, MO, USA) in Dulbecco’s phosphate-buffered saline (DPBS; WELGENE Inc. Gyeongsan-si, Gyeongsangbuk-do, Korea) Stock solutions (100 mM) of compounds **1**–**4** were prepared in dimethyl sulfoxide (DMSO; Sigma-Aldrich).

### 2.2. Determination of ROS

Intracellular ROS levels were measured using the dichlorofluorescin diacetate (DCFDA) assay [39]. Briefly, the cells were seeded in a 96-well plate (1 × 10^4^ cells/well) and continuously starved in serum-free medium during a 24-h incubation. The serum- starved HDFs were treated with 50 and 100 µM of compounds **1**–**4** for 1 h, and then with 20 ng/mL TNF-α. After incubation for 12 h, 10 µM DCFDA (Sigma-Aldrich, St. Louis, MO, USA) was exposed for 15 min and were continuously washed with DPBS. Thereafter, the fluorescence intensity of DCFDA was analyzed using a SPARK 10 M microplate reader (Tecan, Männedorf, Switzerland) at a wavelength of 485 nm.

### 2.3. Determination of Nitric Oxide (NO) Production

Nitric oxide (NO) in cell supernatants was measured using Griess reagent [40]. Briefly, the cells were seeded in a 96-well plate (1 × 10^4^ cells/well) and incubated for 24 h in serum-free medium. The serum-starved HDFs were exposed to 50 and 100 µM of compounds **1**–**4** for 1 h, and then with 20 ng/mL TNF-α for 24 h. Thereafter, the supernatant was collected and homogenized with Griess reagent, and incubated at room temperature. After 10 min, the reaction was analyzed using a SPARK 10 M microplate reader (Tecan, Männedorf, Switzerland) at a wavelength of 540 nm. Sodium nitrite (NaNO_2_) was used for the detection of nitrite concentration (μM).

### 2.4. Determination of Protein Secretion

Production of PGE_2_, MMP-1, procollagen I α1 (COLIA1), IL-1β, IL-6, and IL-8 in the supernatants of cells were measured using an enzyme-linked immunosorbent assay (ELISA) kit [41]. Briefly, the cells were seeded in a 48-well plate (4 × 10^4^ cells/well) and incubated in serum-free medium for 24 h. The serum-starved HDFs were exposed to 50 and 100 µM of the test compounds for 1 h, and then to 20 ng/mL TNF-α for 12 h (IL-1β, IL-6, and IL-8) and 24 h (PGE_2_, MMP-1, and COLIA1). Each supernatant was taken to measure the proinflammatory cytokines, MMP-1, and COLIA1. Concentrations of PGE_2_, MMP-1, COLIA1, IL-1β, IL-6, and IL-8 were measured using the particular ELISA kits according to the protocol (R&D Systems, Minneapolis, MN, USA).

### 2.5. Determination of Gene Expression

The mRNA expression of cells was determined using quantitative real-time polymerase chain reaction (qRT-PCR) [41]. Briefly, the cells were seeded in a 6-well plate (3 × 10^5^ cells/well) and incubated for 24 h. The cells were continuously starved in serum-free medium for 24 h. Thereafter, the HDFs were exposed to 50 and 100 µM compound **1** for 1 h, and then with 20 ng/mL TNF-α for 4 h (IL-1β, IL-6, and IL-8) and 24 h (PGE_2_, MMP-1, and COLIA1). After incubation for the defined time, the cells were washed with DPBS and continuously harvested with lysis buffer for RNA isolation. Total cellular RNA was isolated using the RNeasy Mini Kit (Qiagen, Germantown, MD, USA). Conversion of the isolated RNA to cDNA was accomplished using the RevertAid First Strand cDNA Synthesis kit (Thermo Fisher Scientific, Waltham, MA, USA). qRT-PCR was carried out using the Quant Studio 3 real-time PCR system (Applied Biosystems, Waltham, MA, USA) and PowerUp SYBR PCR Master Mix (Applied Biosystems) at 95 °C for 10 min, followed by 40 cycles of amplification at 95 °C for 1 s and 60 °C for 30 s. The primers used are shown in Table 1. The mRNA expression was normalized and calculated based on β-actin expression and the ratio to 100% of the non-treated group.

### 2.6. Determination of Protein Expression

The protein expression of cells was measured using Western blot analysis [42]. Briefly, the cells were seeded in a 6-well plate (3 × 10^5^ cells/well) and continuously starved in serum-free medium during a 24-h incubation. The serum-starved HDFs were exposed to 50 and 100 µM of compound **1** for 1 h and then to 20 ng/mL TNF-α for 15 min (phos-pho-extracellular signal-regulated kinase [p-ERK], ERK, phospho-C-Jun, T-terminal ki-nase [p-JNK], JNK, p-p38, p38, and glyceraldehyde 3-phosphate dehydrogenase [GAPDH]), 4 h (nuclear factor-kappa B [NF-κB], activator protein-1 [AP-1] and GAPDH), and 6 h (iNOS, COX-2 and GAPDH). Among the proteins described above, the proteins except for GAPDH are activated by TNFα, and occur increasing proinflammatory cytokines and collagenase. GAPDH is housekeeping gene, that was utilized for the normalization of data.

After incubation for each time, the cells were washed with DPBS and continuously harvested with 1× radioimmunoprecipitation assay buffer (RIPA buffer; Tech & Innovation, Gangwon, Korea). Protein concentration was determined using the Pierce™ BCA Protein Assay Kit (Pierce, Rockford, IL, USA).

Equal amounts of protein samples were separated by sodium dodecyl sulfate-polyacrylamide gel electrophoresis (SDS-PAGE) and transferred to polyvinylidene difluoride membrane (Merck Millipore, Darmstadt, Germany). The membranes were blocked in 5% non-fat milk and then the primary antibodies individually added to a membrane and incubated for 4 h at room temperature. The antibodies were directed to iNOS, COX-2, NF-κB (p65), AP-1, ERK1/2, phospho-ERK1/2, p38, phospho-p38, JNK, phospho-JNK, and GAPDH (all from Cell Signaling Technology, Danvers, MA, USA). The antibodies were applied in immunoreaction enhancer solution (Can get signal, Toyobo, Osaka, Japan). After washing, the membranes were incubated with appropriate secondary antibodies (Cell Signaling Technology) for 1 h at room temperature. Subsequently, protein signals were visualized by enhanced chemiluminescence using the Fusion Solo Chemiluminescence System (PEQLAB Biotechnologie GmbH, Erlangen, Germany) and SuperSignal^®^ West Femto Maximum Sensitivity Chemiluminescent Substrate (Pierce). The relative expression of proteins was quantified using ImageJ software (version 1.8.0) from the National Institutes of Health (Bethesda, MD, USA).

### 2.7. Statistical Analyses

The data from the experiments performed in triplicate are expressed as mean ± standard error of the mean (SEM). Statistical analyses were performed using one-way analysis of variance (ANOVA) complemented by the Tukey’s honest significance test. The results were considered statistically significant at *p* < 0.05, *p* < 0.01, and *p* < 0.001.

## 3. Results and Discussion

### 3.1. Effect of Anthraquinones on Production of Intracellular ROS, and Proinflammatory Mediators NO and PGE2 in TNF-α-Stimulated HDFs

In our previous study, four anthraquinones (**1**–**4**) were isolated from an endophytic fungus, *Colletotrichum* sp. JS-0367. Among them, 1,3-dihydroxy-2,8-dimethoxy-6-methylanthraquinone (**1** in Figure 1) and evariquinone (**4** in Figure 1) displayed potent radical scavenging activities of 1,1-diphenyl-2-picrylhydrazyl (DPPH). Compound **4** also displayed neuroprotective effects in glutamate-stimulated murine HT22 hippocampal neuronal cells. The compound was the most potent of the four. It prevented the generation of ROS, intracellular calcium ion levels, and phosphorylation of MAPKs in glutamate-mediated apoptosis [38]. Moreover, many studies have reported that anthraquinones, as antioxidants, have the potential to suppress oxidative stress within cells [43,44,45,46]. Thus, we focused on suppressing the oxidative potential of these anthraquinones in HDFs. In preliminary experiments, the four anthraquinones were not cytotoxic to HDFs at 100 μM (data not shown). 

Therefore, compounds **1**–**4** were expected to have oxidative stress-induced anti-skin aging effects without damage to HDFs. As mentioned above, UV exposure induces intercellular ROS generation and proinflammatory cytokines, such as TNF-α. Furthermore, mitochondrial-derived ROS act as signaling molecules that upregulate inflammatory cytokines, including TNF-α. Excessively increased TNF and ROS regulate increased levels of each other and are activated, triggering diverse inflammatory responses and collagen cleavage. Thus, ROS and TNF-α can be used to test mechanisms similar to those of processes that are induced by UV-induced skin inflammation and aging. We researched the inhibitory effects of anthraquinones **1**–**4** on the production of TNF-α-induced ROS, NO, and PGE_2_.

The change in intracellular ROS levels were measured using DCFDA fluorogenic dye. Serum-starved HDFs were treated with compounds **1**–**4** and subsequently with TNF-α for 12 h. The cells were then exposed to 10 µM DCFDA for 15 min and their fluorescence intensities were measured.

**Figure 2:**
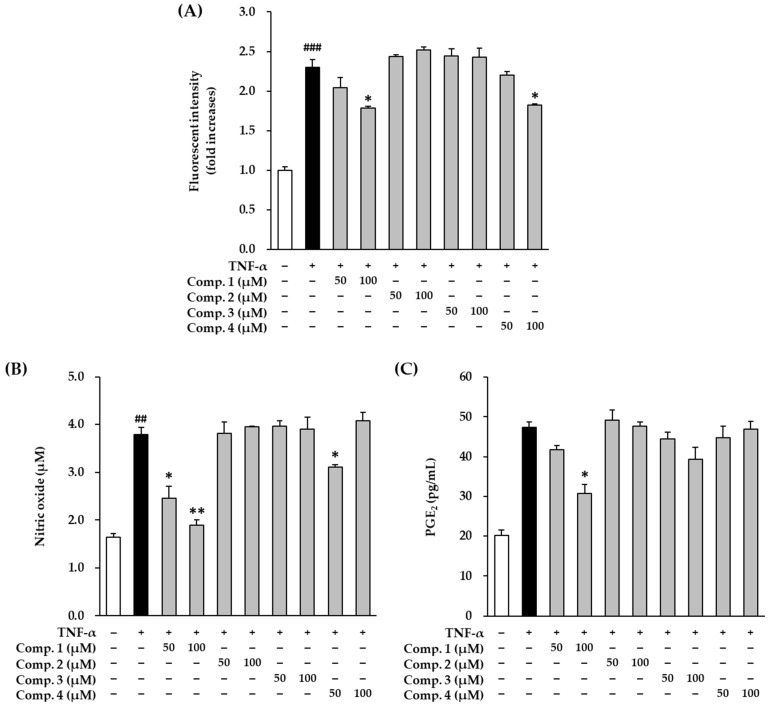
Effects of 1,3-dihydroxy-2,8-dimethoxy-6-methylanthraquinone (**1**), 1-hydroxy-2,3,8- trimethoxy-6-methylanthraquinone (**2**), 1,2-dihydroxy-3,8- dimethoxy-6-methylanthraquinone (**3**) and evariquinone (**4**) on production of intracellular ROS, proinflammatory mediators NO and PGE_2_ in TNF-α-stimulated HDFs. HDFs were untreated or exposed to TNF-α, followed by treatment with **1**–**4** for 24 h. The levels of (**A**) ROS and (**B**) NO and (**C**) PGE_2_ were determined using DCFDA dye, Griess reaction assay, and ELISA. The data are presented as mean ± SEM of at least three independent experiments. ^##^ *p* < 0.01 and ^###^ *p*< 0.001 difference compared to untreated cells. * *p* < 0.05 and ** *p* < 0.01 difference compared to TNF-α-stimulated cells.

TNF-α stimulation increased 2.30 ± 0.03-fold (*p* < 0.01, Figure 2A). It was substantially but non-significantly reduced 1.79 ± 0.02-fold and 1.83 ± 0.01-fold (*p* < 0.05) after treatment with 100 µM of **1** and **4**, respectively. These results showed that ROS generation was suppressed in TNF-α-stimulated HDFs by compounds **1** and **4**. This might be the mechanism involved in ameliorating skin damage induced by oxidative stress.

The four compounds are presented typical anthraquinone structure. In moieties of anthraquinone, they equally attached with the one hydroxyl group, the one methyl group and the one methoxy group to carbon positions 1, 6 and 8 (C-1, 6, 8), respectively. They are substituted the hydroxy groups or the methoxy groups at C-2, 3, but their numbers and positions are different. In detail, compound **1** was attached with the one hydroxyl groups at C-3 and the one methoxy groups at C-2. Differently, compound **3** was attached at changed positions, that with the one hydroxyl group at C-2, and the one methoxy groups at C-3. Compounds **2** and **4** were bound to only one functional group, the two methoxy groups or the two hydroxyl groups at C-2 and 3. In ROS generation, compounds 1 and 4 were presented with EC_20_ (concentration of compound that produces 20% biological effect) of 51.1 and 71.2 µM, respectively, whereas compounds **2** and **3** were not shown below 100 µM (Tables S1–S6). These results indicate that the mechanism of ROS scavenging by anthraquinone, may depend on the correlation between the hydroxyl group at C-3 and the ketone group at C-10).

TNF-α stimulation significantly increased the production of nitric oxide (NO) from 1.65 ± 0.07 (*p* < 0.01) to 3.79 ± 0.15 µM (*p* < 0.01) (Figure 2B). NO levels were significantly reduced to 2.46 ± 0.25 (*p* < 0.05) and 1.90 ± 0.10 µM (*p* < 0.01) after treatment with 50 and 100 µM of compound **1**, respectively. Compound **4** also reduced NO to 3.12 ± 0.04 µM (*p* < 0.05) after treatment with 50 µM. However, NO levels tended to increase using 100 µM of **4** (4.09 ± 0.17 µM). Compounds **2** and **3** did not reduce NO production. For NO production, EC_50_ (concentration of compound that produces 50% biological effect) of compound **1** was 27.0 µM, while other compounds not showed significant EC_50_ below 100 µM (Tables S1–S6). These results show that the NO inhibition by anthraquinone, also possibly depend on the correlation between the hydroxyl group at C-3 and the ketone group at C-10).

TNF-α treatment remarkably increased the levels of PGE_2_ from 20.3 ± 0.10 pg/mL to 47.4 ± 1.33 pg/mL (Figure 2C). Compound **1** treatment dramatically reduced the increased PGE_2_ levels in a dose-dependent manner (50 µM; 41.8 ± 0.98 pg/mL, 100 µM; 31.8 ± 2.93 pg/mL, *p* < 0.05). Compound **3** treatment also suppressed the increased PGE_2_ levels in a dose-dependent manner (50 µM; 45.5.8 ± 1.64 pg/mL, 100 µM; 39.4 ± 3.05 pg/mL). Compounds **2** and **4** did not inhibit PGE_2_ production. In PGE_2_ generation, compounds **1** and **3** were showed with EC_20_ (concentration of compound that produces 20% biological effect) of 49.3 and 75.3 µM, respectively, whereas compounds **2** and **4** were not significant EC_50_ below 100 µM (Table S1). The mechanism of PGE_2_ production was also expected to be related to the hydroxyl group of C-3 and the ketone group of C-10, but it was not clear because other differences were also discovered.

Taken together, these results indicate that compound **1** potently scavenges excess ROS and suppresses the production of NO and PGE_2_, compared with compounds **2**–**4**. For this reason, we subsequently focused on compound **1**.

### 3.2. Effect of Compound ***1*** on COX-2 and iNOS Expression in TNF-α-Stimulated HDFs

The activation of COX-2 and iNOS is important role in NO and PGE_2_ production. We investigated the effect of compound **1** on the protein expression of COX-2 and iNOS by serum-starved TNF-α-stimulated HDFs.

**Figure 3:**
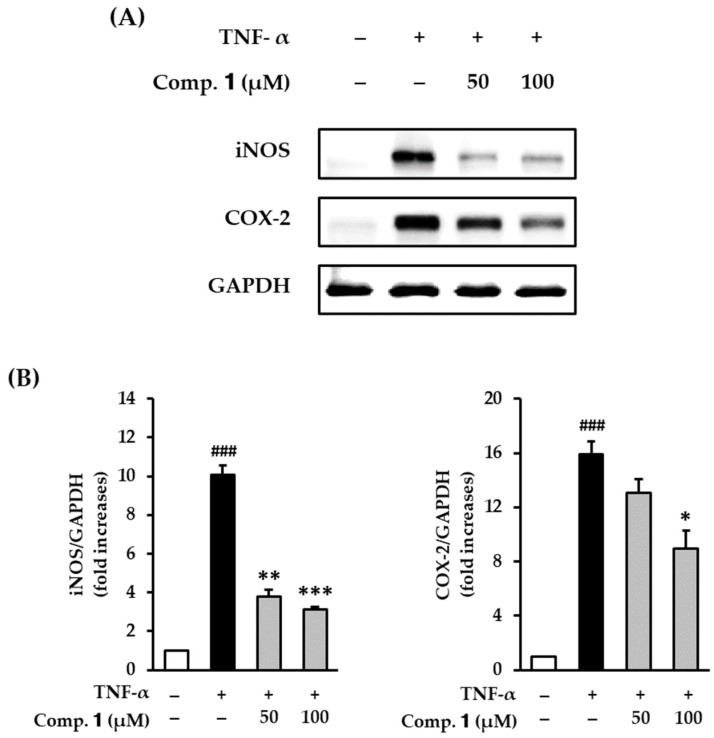
Effects of 1,3-dihydroxy-2,8-dimethoxy-6-methylanthraquinone (**1**) on COX-2 and iNOS protein expression in TNF-α-stimulated HDFs. HDFs were untreated or treated with TNF-α, followed by treatment with **1** for 6 h. The levels of protein expression were determined using Western blot analysis. (**A**) The protein expression of iNOS, COX-2, and GAPDH. (**B**) The graphs of relative protein expression levels of iNOS and COX-2. The data are presented as mean ± SEM of at least three independent experiments. ^###^ *p* < 0.001 difference compared to untreated cells. * *p* < 0.05, ** *p* < 0.01 and *** *p* < 0.001 difference compared to TNF-α-stimulated cells.

After TNF-α treatment, expression of both iNOS (10.1 ± 0.51-fold, *p* < 0.001) and COX-2 (15.9 ± 1.00-fold, *p* < 0.001) expressions were significantly increased compared with the untreated group. The increased activities were inhibited by compound **1** (Figure 3A) in a dose-dependent manner (Figure 3B). The protein expression of iNOS was significantly reduced to 3.78 ± 0.34 (*p* < 0.05) and 3.10 ± 0.17-fold (*p* < 0.01) by 50 and 100 µM of compound **1**, respectively. The protein expression of COX-2 was also reduced to 13.0 ± 1.06 (not significant) and 8.96 ± 1.33-fold (*p* < 0.05) by 50 and 100 µM of compound **1**, respectively. These results indicate that compound **1** may inhibit inflammation in TNF-α-stimulated HDFs.

Previous studies have demonstrated that anthraquinones inhibit inflammatory mediators (NO and COX-2) and suppress inflammatory responses via the NF-κB pathway [47,48]. Consistent with the prior data, compound **1** also suppressed the inflammatory response to iNOS and COX-2 in TNF-α-stimulated HDFs. Therefore, compound **1** can ameliorate inflammation induced by the generation of ROS.

### 3.3. Effect of Compound ***1*** on MMP-1 and COLIA1 mRNA in TNF-α-Stimulated HDFs

The ECM of skin is a complex collection of collagen and non-collagen components. The generation of ROS is induced by external stimuli that include UV radiation. ROS alters the gene and protein structure, including collagen and collagen-degrading enzymes. Ultimately, the changes damage the skin ECM, leading to aging related features like wrinkles [5,49]. MMP-1 is a collagenase that plays a critical role in the degradation of collagen in the skin. Therefore, inhibitors of MMP-1 activity may be a potential candidate for anti-skin aging, such as wrinkle formation [50]. We investigated MMP-1 expression in TNF-α-stimulated HDFs.

**Figure 4:**
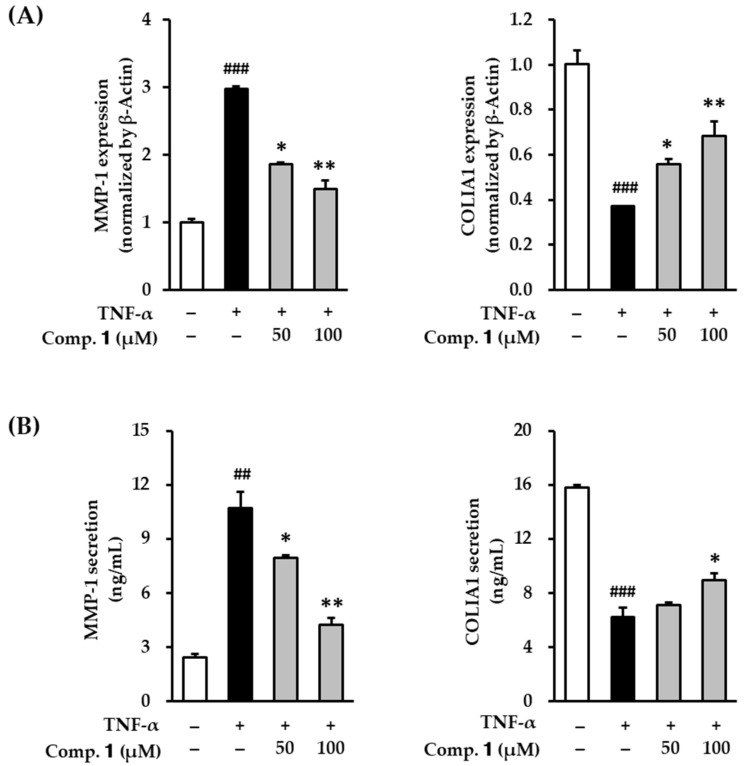
Effects of 1,3-dihydroxy-2,8-dimethoxy-6-methylanthraquinone (**1**) on MMP-1 and COLIA1 mRNA and protein expression in TNF-α-stimulated HDFs. HDFs were untreated or exposed to TNF-α, followed by treated with **1** for 4 h or 12 h. The levels of mRNA and protein expression were determined using qRT-PCR and ELISA. (**A**) Relative mRNA expression levels of *MMP-1* and *COLIA1.* (**B**) Protein expression of MMP-1 and COLIA1. The data are presented as mean ± SEM from three independent experiments. ^##^ *p* < 0.01 and ^###^ *p* < 0.001 difference compared to untreated cells. * *p* < 0.05 and ** *p* < 0.01 difference compared to TNF-α-stimulated cells.

Compound **1** significantly suppressed the mRNA expression and protein secretion of the MMP-1 collagenase in the skin ECM (Figure 4). As shown in Figure 4A, TNF-α treatment significantly increased the mRNA expression of MMP-1 to 2.98 ± 0.04-fold compared with the untreated group. TNF-α expression was significantly decreased to 1.86 ± 0.03-fold (*p* < 0.05) and 1.49 ± 0.13-fold (*p* < 0.01) by 50 and 100 µM of compound **1**, respectively. Analogously, TNF-α treatment also increased the protein secretion of MMP-1 to 10.7 ± 0.90 ng/mL compared with the untreated group (2.43 ± 0.22 ng/mL). It was significantly decreased to 7.95 ± 0.15 (*p* < 0.05) and 4.25 ± 0.39 ng/mL (*p* < 0.01) by 50 and 100 µM of compound **1**, respectively. These results demonstrate that compound **1** can suppress both gene expression and protein secretion of collagenase MMP-1 in TNF-α-stimulated HDFs.

Collagen was synthesized from procollagen, which is a precursor molecule containing additional peptide sequences. Because these sequences are cleaved during collagen secretion, several sequences have indirect information about collagen synthesis levels. Thus, we determined COLIA1 to investigate collagen synthesis. As shown in Figure 4A, TNF-α treatment significantly decreased the mRNA expression of COLIA1 to 0.37 ± 0.00-fold compared with the untreated group. It was significantly increased to 0.56 ± 0.03-fold (*p* < 0.05) and 0.69 ± 0.06-fold (*p* < 0.01) by 50 and 100 µM of compound **1**, respectively. Analogously, TNF-α treatment also reduced the protein secretion of COLIA1 to 6.25 ± 0.70 ng/mL compared with untreated group (15.8 ± 0.20 ng/mL). COLIA1 was decreased to 7.09 ± 0.22 (not significant) and 8.95 ± 0.48 ng/mL (*p* < 0.01) by 50 and 100 µM of compound **1**, respectively. These results demonstrate that compound **1** increased both gene expression and protein of procollagen in TNF-α-stimulated HDFs. Therefore, compound 1 might potentially enhance of skin ECM degradation by oxidative stress.

### 3.4. Effect of Compound ***1*** on Proinflammatory Cytokines in TNF-α-Stimulated HDFs

Cellular oxidative stress produces proinflammatory cytokines, such as TNF-α, IL-1β, IL-6, and IL-8, and is involved in the upregulation of the inflammatory response [51,52]. These inflammatory responses cause skin aging and diverse cutaneous lesions [53,54]. To assess the inhibitory effect of compound **1** on the inflammatory response in skin dermal cells, we verified the effect of compound **1** on *IL-1β*, *IL-6,* and *IL-8* mRNA expression in TNF-α-stimulated HDFs. To investigate whether compound **1** inhibited the inflammatory response in skin cells, we directly determined mRNA gene expression of *IL-1β*, *IL-6*, and *IL-8* in TNF-α-stimulated HDFs.

**Figure 5:**
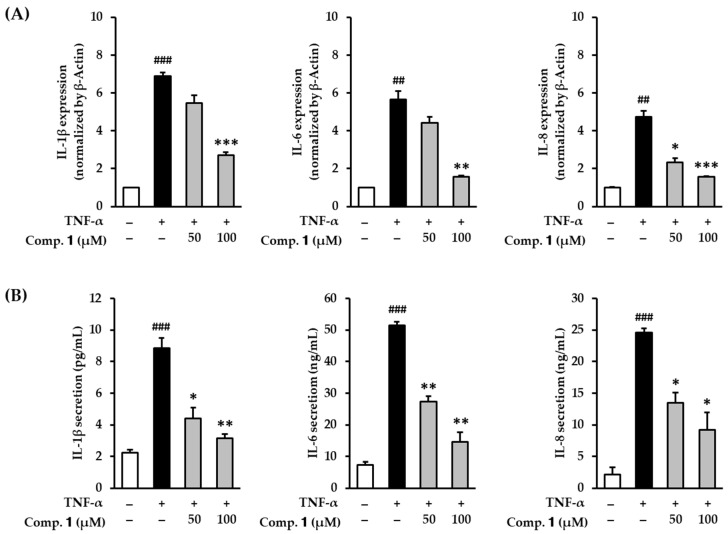
Effects of 1,3-dihydroxy-2,8-dimethoxy-6-methylanthraquinone (**1**) on mRNA and protein expression of proinflammatory cytokines IL-1β, IL-6, and IL-8 in TNF-α-stimulated HDFs. HDFs were untreated or treated with TNF-α, followed by treatment with **1** for 4 h or 12 h. The mRNA and protein expression levels were determined using qRT-PCR and ELISA. (**A**) Relative mRNA expression levels of *IL-1β*, *IL-6*, and *IL-8*. (**B**) Protein expression of IL-1β, IL-6, and IL-8. The data are presented as mean ± SEM from three independent experiments. ^##^ *p* < 0.01 and ^###^ *p* < 0.001 difference compared to untreated cells. * *p* < 0.05, ** *p* < 0.01, *** *p* < 0.001 difference compared to TNF-α-stimulated cells.

Serum-starved HDFs were challenged with compound **1** followed by TNF-α for 4 h. Next, mRNA expression was determined by qRT-PCR. As shown in Figure 5A, TNF-α treatment clearly increased the mRNA expression of *IL-1β* to 6.90 ± 0.19-fold compared with the untreated group. *IL-1β* expression was clearly decreased to 2.71 ± 0.15-fold (*p* < 0.05) by 100 µM of compound **1**. Analogously, TNF-α treatment significantly increased the mRNA expression of *IL-6* to 5.66 ± 0.45-fold compared with the untreated group. The expression was significantly reversed to 1.58 ± 0.06-fold (*p* < 0.01) by compound **1**. TNF-α stimulation increased *IL-8* mRNA expression to 4.75 ± 0.31-fold compared with non-treated cells. The expression was concentration-dependently suppressed by compound **1** (50 μM; 2.33 ± 0.23-fold, *p* < 0.05, 100 μM; 1.57 ± 0.04-fold, *p* < 0.001). These results mean that compound 1 raised the gene expression of proinflammatory cytokines in TNF-α-stimulated HDFs.

Additionally, to measure the change in the actual secretion of inflammatory cytokines, we performed ELISA. As shown Figure 5B, TNF-α stimulation increased IL-1β from 2.23 ± 0.18 to 8.84 ± 0.66 pg/mL (*p* < 0.001), IL-6 from 7.45 ± 0.95 to 51.6 ± 1.05 ng/mL (*p* < 0.001), and IL-8 from 2.19 ± 1.16 to 24.6 ± 0.63 ng/mL (*p* < 0.001) expression. The secretion of IL-1β was significantly reduced to 4.42 ± 0.66- (*p* < 0.05) and 3.13 ± 0.27-fold (*p* < 0.01) by 50 and 100 µM of compound **1**, respectively. The secretion of IL-6 was significantly reduced to 27.4 ± 1.68-fold (*p* < 0.01) and 14.6 ± 3.05-fold (*p* < 0.01) by 50 and 100 µM of compound **1**, respectively. The secretion of IL-8 was significantly reduced to 13.5 ± 1.68-fold (*p* < 0.05) and 9.24 ± 2.74-fold (*p* < 0.05) by 50 and 100 µM of compound **1**, respectively. Consistent with mRNA expression, the protein levels of proinflammatory cytokines were significantly reduced in TNF-α-stimulated HDFs. These results indicate that compound **1** may suppress skin inflammatory responses stimulated by TNF-α by inhibiting proinflammatory cytokines. Therefore, compound **1** may potentially help lessen inflammation-related skin aging and diseases.

### 3.5. Effect of Compound ***1*** on NF-κB and AP-1 Expression in TNF-α-Stimulated HDFs

MMP-1 and proinflammatory cytokine levels are upregulated by the AP-1 and NF-κB. Thus, compound **1** might promote collagen synthesis by inhibiting MMP-1 and proinflammatory cytokine expression levels. Western blot analysis was conducted to further investigate the role of the AP-1 and NF-κB in the action of compound **1**.

**Figure 6:**
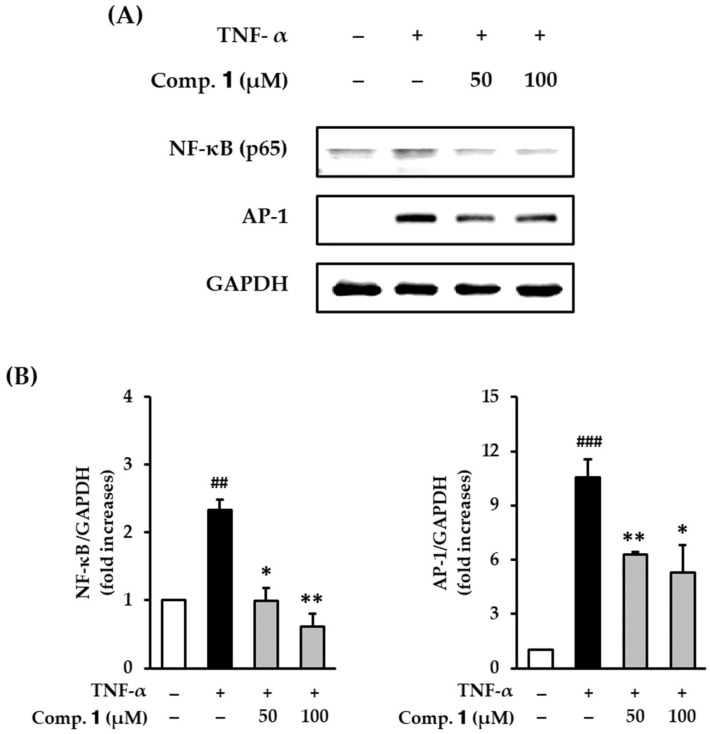
Effects of 1,3-dihydroxy-2,8-dimethoxy-6-methylanthraquinone (**1**) on NF-κB and AP-1 protein expression in TNF-α-stimulated HDFs. HDFs were untreated or treated with TNF-α, followed by treatment with **1** for 12 h. Protein levels were determined using Western blot analysis. (**A**) Protein expression of NF-κB, AP-1, and GAPDH. (**B**) Relative protein expression levels of NF-κB and AP-1. The data are presented as mean ± SEM of from three independent experiments. ^##^ *p* < 0.01 and ^###^ *p* < 0.001 difference compared to untreated cells. * *p* < 0.05 and ** *p* < 0.01 difference compared to TNF-α-stimulated cells.

Serum-starved HDFs were treated with compound **1**, followed by TNF-α for 6 h. Protein expression was measured by Western blot analysis. TNF-α treatment significantly increased the expression of both NF-κB (p65) (2.34 ± 0.15-fold, *p* < 0.01) and AP-1 (10.6 ± 1.02-fold, *p* < 0.001) (Figure 6A). Compound **1** concentration-dependently inhibited TNF-α-stimulated NF-κB (p65) and AP-1 expressions (Figure 6B). The protein expression of NF-κB (p65) was significantly reduced to 1.00 ± 0.18-fold (*p* < 0.05) and 0.62 ± 0.19-fold (*p* < 0.01) by 50 and 100 µM of compound **1**, respectively. Analogously, the protein expressions of AP-1 also were substantially reduced to 6.31 ± 0.12-fold (*p* < 0.01) and 5.27 ± 1.55-fold (*p* < 0.05) by 50 and 100 µM of compound **1**, respectively. These results indicate that compound **1** can suppress the skin inflammatory response induced by TNF-α stimulation by regulating AP-1 and NF-κB activation. These results demonstrated that compound 1 may suppress skin inflammatory responses stimulated by TNF-α by down-regulating AP-1 and NF-κB activation.

### 3.6. Effect of Compound ***1*** on TNF-α-Stimulated Phosphorylation of MAPKs in HDFs

AP-1 and NF-κB pathways regulate MMP-1 and proinflammatory cytokines. The pathways are regulated by signaling of MAPKs. To determine whether compound **1** could inhibit MAPK phosphorylation in TNF-α stimulation, we investigated the effects of compound **1** on TNF-α-stimulated phosphorylation of MAPKs in HDFs. Serum-starved HDFs were challenged with compound **1** followed by TNF-α for 15 min. Expression of protein was measured by Western blotting.

**Figure 7:**
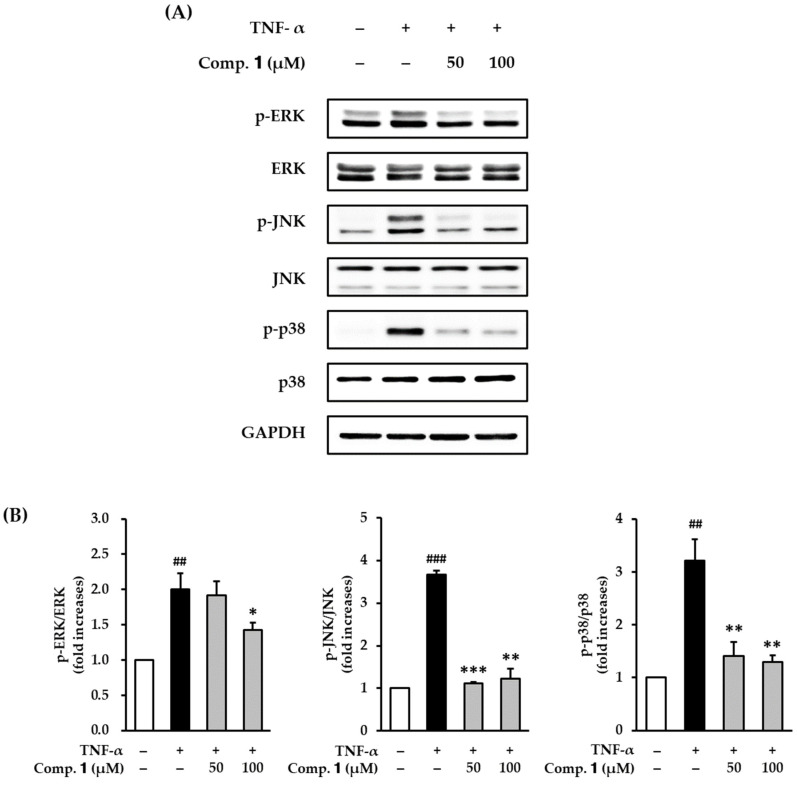
Effects of 1,3-dihydroxy-2,8-dimethoxy-6-methylanthraquinone (**1**) on phosphorylation of MAPKs in TNF-α-stimulated HDFs. HDFs were untreated or treated with TNF-α, followed by treatment with **1** for 15 min. Protein expression levels were determined using Western blot analysis. (**A**) Protein expressions of p-ERK, ERK, p-JNK, JNK, p-p38, p38, and GAPDH. (**B**) Relative protein expression levels of p-ERK/ERK, p-JNK/JNK, p-p38/p38, and GAPDH. The data are presented as mean ± SEM from three independent experiments. ^##^ *p* < 0.01 and ^###^ *p* < 0.001 difference compared to untreated cells. * *p* < 0.05, ** *p* < 0.01, *** *p* < 0.001 difference compared to TNF-α-stimulated cells.

Phosphorylation of MAPKs, including ERK, JNK, and p38, was increased in the TNF-α-stimulated HDFs, and was clearly inhibited by compound **1** treatment (Figure 7). The ratio of p-ERK/ERK in the TNF-α- group was 2.01 ± 0.22-fold higher than the non-treated group, it was dramatically decreased to 1.43 ± 0.10-fold (*p* < 0.05) by treatment with 100 µM (Figure 7B). The phosphorylation of JNK was clearly increased in the TNF-α group to 2.67 ± 0.08-fold compared with the non-treated group, and it was clearly decreased to 1.11 ± 0.03- (50 µM, *p* < 0.001) and 1.23 ± 0.23-fold (100 µM; *p* < 0.01) by compound **1** treatment. Similarly, the phosphorylation of p38 was substantially increased to 3.21 ± 0.40-fold by TNF-α stimulation, and was significantly decreased to 1.41 ± 0.26- (50 µM, *p* < 0.01) and 1.29 ± 0.12-fold (100 µM; *p* < 0.01) by treatment with compound **1**. These results demonstrated that compound **1** may suppress AP-1 and NF-κB activation, which is stimulated by TNF-α, by suppressing the phosphorylation of MAPKs.

UV can directly or indirectly induce ROS and several proinflammatory mediators, such as PGE_2_, COX-2, iNOS, IL-1β, IL-6, and IL-8 [15,16,17,18,19]. These molecules are related to skin damage and accelerate photoaging induced by UV irradiation [17,20]. Several studies have demonstrated that antioxidants inhibit ROS generation, leading to the prevention of skin photoaging, and that ROS upregulates the activation of AP-1, NF-κB, and MAPKs [55,56,57]. The phosphorylations of p38, ERK, and JNK induce the excessive synthesis of the MMP-1 collagenase [58,59,60]. TNF-α-induced ROS generation causes degradation of the skin ECM due to the activation of AP-1, NF-κB, and MAPKs [53,54] Thus, compound **1**-dependent inhibition of AP-1, NF-κB, and MAPKs activation may account for the ability of this compound to attenuate inflammatory response and MMP-1 synthesis. 

In summary, 1,3-dihydroxy-2,8-dimethoxy-6-methylanthraquinone (**1**) isolated from the endophytic fungus, *Colletotrichum* sp. JS-0367 has antioxidant and inflammatory effects due to the inhibition of intracellular ROS, NO, and PGE_2_ generation in TNF-α-stimulated HDFs. Compound **1** can prevent degradation of the skin ECM due to increased MMP-1 collagenase and decreased collagen synthesis. Mechanistically, compound 1 suppresses iNOS and COX-2 activation and the proinflammatory cytokines IL-1β, IL-6, and IL-8. The compound **1**-mediated inhibition of TNF-α-stimulated skin aging in HDFs is related to the inhibition of NF-κB, AP-1, and MAPKs activation.

## 4. Conclusions

ROS are major causative factors of inflammatory responses and ECM degradation. ROS cause skin aging and diverse cutaneous lesions. Thus, ROS inhibitors may lessen skin aging and diseases. The present data demonstrate that 1,3-dihydroxy-2,8-dimethoxy-6-methylanthraquinone (**1**), a novel anthraquinone isolated from *Colletotrichum* sp. JS-0367 reduces TNF-α-stimulated ROS, NO, and PGE_2_, attenuated MMP-1 expression, and enhances collagen synthesis. Furthermore, compound **1** inhibits the expression of TNF-α-stimulated proinflammatory cytokine mediators, including iNOS and COX-2, and proinflammatory cytokines IL-1, IL-6, and IL-8. The mechanism by which compound 1 inhibits TNF-α-stimulated skin aging in HDFs involves the inhibition of NF-κB, AP-1, and MAPKs activation. The present data provide the potent evidence that compound **1** may be beneficial in improving skin damage. Although more extensive studies are needed for a thorough understanding of the protective effects of compound **1** on skin aging, the compound is a potential candidate for improving skin aging and diverse cutaneous lesions.

## Figures and Tables

**Table 1:** Primer sequences.

Genes	Sequences	
*Matrix metalloproteinase-1* (AF158733)	Sense	5′- ATTCTACTGATATCGGGGCTTT -3′
	Antisense	5′- ATGTCCTTGGGGTATCCGTGTA -3′
*Procollagen I α1* (X07884)	Sense	5′-CTCGAGGTGGACACCACCCT-3′
	Antisense	5′-CAGCTGGATGGCCACATCGG-3′
*Interleukin-1β* (NM_000576)	Sense	5′-CTGTCCTGCGTGTTGAAAGA-3′
	Antisense	5′-TTCTGCTTGAGAGGTGCTGA-3′
*Interleukin-6* (HUMIL6CSF)	Sense	5′-CAGGAATTGAATGGGTTTGC-3′
	Antisense	5′-AAACCAAGGCACAGTGGAAC-3′
*Interleukin-8* (HUMIL8A)	Sense	5′-CTCCTTCTCCACAAGCGCC-3′
	Antisense	5′-GCCGAAGAGCCCTCAGGC-3′
*β-Actin* (DQ407611)	Sense	5′-AGAGATGGCCACGGCTGCTT-3′
	Antisense	5′-ATTTGCGGTGGACGATGGAG-3′

## Data Availability

Data sharing not applicable.

## Supplementary Materials

The following are available online at https://www.mdpi.com/2076-3921/10/2/200/s1, Table S1: Effects of 1,3-dihydroxy-2,8-dimethoxy-6-methylanthraquinone (**1**), 1-hydroxy-2,3,8- trimethoxy-6-methylanthraquinone (**2**), 1,2-dihydroxy-3,8- dimethoxy-6-methylanthraquinone (**3**) and evariquinone (**4**) on production of intracellular ROS, proinflammatory mediators NO and PGE_2_ in TNF-α-stimulated HDFs; Table S2: The relative protein expression of iNOS, COX-2. Effects of 1,3-dihydroxy-2,8-dimethoxy-6-methylanthraquinone (**1**) on COX-2 and iNOS protein expression in TNF-α-stimulated HDFs; Table S3: Relative mRNA and protein expression of MMP-1 and COLIA1. Effects of 1,3-dihydroxy-2,8-dimethoxy-6-methylanthraquinone (**1**) on MMP-1 and COLIA1 mRNA and protein expression in TNF-α-stimulated HDFs; Table S4: Relative mRNA and protein expression of IL-1β, IL-6, and IL-8. Effects of 1,3-dihydroxy-2,8-dimethoxy-6-methylanthraquinone (**1**) on mRNA and protein expression of proinflammatory cytokines IL-1β, IL-6, and IL-8 in TNF-α-stimulated HDFs; Table S5: Relative protein expression of NF-κB and AP-1. Effects of 1,3-dihydroxy-2,8-dimethoxy-6-methylanthraquinone (**1**) on NF-κB and AP-1 protein expression in TNF-α-stimulated HDFs; Table S6: Relative protein expression of p-ERK, ERK, p-JNK, JNK, p-p38, and p38. Effects of 1,3-dihydroxy-2,8-dimethoxy-6-methylanthraquinone (**1**) on phosphorylation of MAPKs in TNF-α-stimulated HDFs.
