# Supplementary material for: Preventive Effects of Anthraquinones Isolated from an Endophytic Fungus, Colletotrichum sp. JS-0367 in Tumor Necrosis Factor-α-Stimulated Damage of Human Dermal Fibroblasts
Source: Antioxidants (Basel). 2021 Jan 30;10(2):200. doi: 10.3390/antiox10020200 (PMC7910856; doi:10.3390/antiox10020200)
Supplement: Supplementary file 1 [file antioxidants-10-00200-s001.pdf]

antioxidants

MDPI

Supplementary material

Preventive Effects of Anthraquinones Isolated from an Endophytic Fungus, *Colletotrichum* sp. JS-0367 in Tumor Necrosis Factor-α-Stimulated Damage of Human Dermal Fibroblasts

Sullim Lee [1], Quynh Nhu Nguyen [2], Hung Manh Phung [2], Sang Hee Shim [3], Daeyoung Kim [1], Gwi Seo Hwang [2] and Ki Sung Kang [2,*]

[1] Department of Life Science, College of Bio-Nano Technology, Gachon University, Seongnam 13120, Korea; sullimlee@gachon.ac.kr (S.L.); davekim@gachon.ac.kr (D.K.)
[2] College of Korean Medicine, Gachon University, Seongnam 13120, Korea; nnquynh173@gmail.com (Q.N.N.); 201940218@gc.gachon.ac.kr (H.M.P.), seoul@gachon.ac.kr (G.S.H.)
[3] College of Pharmacy, Duksung Women's University, Seoul 01369, Korea; sangheeshim@duksung.ac.kr
* Correspondence: kkang@gachon.ac.kr; Tel.: +82-31-750-5402; Fax: +82-31-750-5416

Supplementary table S1. Effects of 1,3-dihydroxy-2,8-dimethoxy-6-methylanthraquinone (**1**), 1-hydroxy-2,3,8- trimethoxy-6-methyl-anthraquinone (**2**), 1,2-dihydroxy-3,8- dimethoxy-6-methylanthraquinone (**3**) and evariquinone (**4**) on production of intracellular ROS, proinflammatory mediators NO and PGE_2 in TNF-α-stimulated HDFs. HDFs were untreated or exposed to TNF-α, followed by treatment with **1** - **4** for 24 h. The levels of (A) ROS and (b) NO and PGE_2 were determined using DCFDA dye, Griess reaction assay, and ELISA. The data are presented as mean ± SEM of at least three independent experiments. (EC_{20} : concentration of compound that produces 20% biological effect, EC_{50} : concentration of compound that produces 50% biological effect)

TNFα	Samples	Concentration (μM)	Fluorescent intensity (fold increases)	EC_{20} (μM)	NO (μM)	EC_{50} (μM)	PGE_2 (pg/mL)	EC_{20} (μM)
-	-	-	1.00 ± 0.05		1.65 ± 0.07		20.2 ± 1.29	
+	-	-	2.30 ± 0.10		3.79 ± 0.15		47.4 ± 1.33	
+	Comp. **1**	50	2.05 ± 0.13	51.1	2.46 ± 0.25	27.0	41.8 ± 0.98	49.5
+	Comp. **1**	100	1.79 ± 0.02		1.90 ± 0.10		30.8 ± 2.30	
+	Comp. **2**	50	2.43 ± 0.02	>100	3.81 ± 0.25	>100	49.2 ± 2.53	>100
+	Comp. **2**	100	2.52 ± 0.04		3.96 ± 0.01		47.7 ± 0.95	
+	Comp. **3**	50	2.45 ± 0.09	>100	3.97 ± 0.12	>100	44.5 ± 1.64	75.3
+	Comp. **3**	100	2.43 ± 0.11		3.90 ± 0.25		39.4 ± 3.05	
+	Comp. **4**	50	2.20 ± 0.05	71.2	3.12 ± 0.04	-	44.9 ± 2.75	-
+	Comp. **4**	100	1.83 ± 0.01		4.09 ± 0.17		46.8 ± 2.07	

Supplementary table S2. The relative protein expression of iNOS, COX-2. Effects of 1,3-dihydroxy-2,8-dimethoxy-6-methylanthraquinone (**1**) on COX-2 and iNOS protein expression in TNF-α-stimulated HDFs. HDFs were untreated or treated with TNF-α, followed by treatment with **1** for 6 h. The levels of protein expression were determined using western blot analysis. The data are presented as mean ± SEM of at least three independent experiments.

**TNFα**	**Comp.1 (μM)**	**iNOS/GAPDH (fold increases)**			**COX-2/GAPDH (fold increases)**		
-	-	1.00	±	0.00	1.00	±	0.00
+	-	10.1	±	0.51	15.9	±	1.00

+	50	3.78	±	0.34	13.1	±	1.06
+	100	3.10	±	0.17	8.96	±	1.33

Supplementary table S3. Relative mRNA and protein expression of MMP-1 and COLIA1. Effects of 1,3-dihydroxy-2,8-dimethoxy-6-methylanthraquinone (**1**) on MMP-1 and COLIA1 mRNA and protein expression in TNF-α-stimulated HDFs. HDFs were untreated or exposed to TNF-α, followed by treated with **1** for 4 h or 12 h. The levels of mRNA and protein expression were determined using qRT-PCR and ELISA. The data are presented as mean ± SEM from three independent experiments.

TNFα	Comp.1 (μM)	MMP-1 expression (normalized by β-Actin)			COLIA1 expression (normalized by β-Actin)			MMP-1 secretion (ng/mL)			COLIA1 secretion (ng/mL)		
-	-	1.00	±	0.05	1.00	±	0.06	2.43	±	0.22	15.8	±	0.20
+	-	2.98	±	0.04	0.37	±	0.00	10.7	±	0.90	6.25	±	0.70
+	50	1.86	±	0.03	0.56	±	0.03	7.95	±	0.15	7.09	±	0.22
+	100	1.49	±	0.13	0.69	±	0.06	4.25	±	0.39	8.95	±	0.48

Supplementary table S4. Relative mRNA and protein expression of IL-1β, IL-6, and IL-8. Effects of 1,3-dihydroxy-2,8-dimethoxy-6-methylanthraquinone (**1**) on mRNA and protein expression of proinflammatory cytokines IL-1β, IL-6, and IL-8 in TNF-α-stimulated HDFs. HDFs were untreated or treated with TNF-α, followed by treatment with **1** for 4 h or 12 h. The mRNA and protein expression levels were determined using qRT-PCR and ELISA. The data are presented as mean ± SEM from three independent experiments.

TNFα	Comp.1 (μM)	IL-1β expression (normalized by β-Actin)			IL-6 expression (normalized by β-Actin)			IL-8 expression (normalized by β-Actin)		
-	-	1.00	±	0.01	1.00	±	0.01	1.00	±	0.03
+	-	6.90	±	0.19	5.66	±	0.45	4.75	±	0.31
+	50	5.46	±	0.42	4.43	±	0.29	2.33	±	0.23
+	100	2.71	±	0.15	1.58	±	0.06	1.57	±	0.04
		**IL-1β secretion (pg/mL)**			**IL-6 secretion (ng/mL)**			**IL-8 secretion (pg/mL)**		
-	-	2.25	±	0.18	7.45	±	0.95	2.19	±	1.16
+	-	8.84	±	0.66	51.6	±	1.05	24.6	±	0.63
+	50	4.42	±	0.66	27.4	±	1.68	13.5	±	1.68
+	100	3.13	±	0.27	14.6	±	3.05	9.24	±	2.74

Supplementary table S5. Relative protein expression of NF-κB and AP-1. Effects of 1,3-dihydroxy-2,8-dimethoxy-6-methylanthraquinone (**1**) on NF-κB and AP-1 protein expression in TNF-α-stimulated HDFs. HDFs were untreated or treated with TNF-α, followed by treatment with **1** for 12 h. Protein levels were determined using western blot analysis. The data are presented as mean ± SEM of from three independent experiments.

TNFα	Comp.1 (μM)	NF-κB/GAPDH (fold increases)			AP-1/GAPDH (fold increases)		
-	-	1.00	±	0.03	1.00	±	0.09
+	-	2.34	±	0.15	10.6	±	1.02
+	50	1.00	±	0.18	6.31	±	0.12
+	100	0.62	±	0.19	5.27	±	1.55

Supplementary table S6. Relative protein expression of p-ERK, ERK, p-JNK, JNK, p-p38, and p38. Effects of 1,3-dihydroxy-2,8-dimethoxy-6-methylanthraquinone (**1**) on phosphorylation of MAPKs in TNF-α-stimulated HDFs. HDFs were untreated or treated with TNF-α, followed by treatment with **1** for 15 min. Protein expression levels were determined using western blot analysis. The data are presented as mean ± SEM from three independent experiments.

TNFα	Comp.1 (μM)	p-ERK/ERK (fold increases)			p-JNK/JNK (fold increases)			p-p38/p38 (fold increases)		
-	-	1.00	±	0.10	1.00	±	0.12	1.00	±	0.06
+	-	2.01	±	0.22	3.67	±	0.10	3.21	±	0.40
+	50	1.92	±	0.20	1.11	±	0.03	1.41	±	0.26
+	100	1.43	±	0.10	1.23	±	0.23	1.29	±	0.12
